# Survival Mechanisms and Influence Factors of Circulating Tumor Cells

**DOI:** 10.1155/2018/6304701

**Published:** 2018-11-01

**Authors:** Wen-Chao Wang, Xiao-Feng Zhang, Jian Peng, Xi-Feng Li, Ai-Li Wang, Ya-Qin Bie, Le-Hua Shi, Mou-Bin Lin, Xiao-Feng Zhang

**Affiliations:** ^1^Yangpu Hospital, Tongji University School of Medicine, Shanghai 200090, China; ^2^Department of Hepatic Surgery IV, The Eastern Hepatobiliary Surgery Hospital, Second Military Medical University, Shanghai 200438, China; ^3^Molecular Oncology Laboratory, The Eastern Hepatobiliary Surgery Hospital, Second Military Medical University, Shanghai 200438, China

## Abstract

Circulating tumor cells (CTCs) are cancer cells shed from either the primary tumor or its metastases that circulate in the peripheral blood. The CTCs are regarded as the source of tumor recurrence and metastasis and speculated as the indicators of residual tumors, thereby indicating a poor prognosis. Although CTCs play a vital role in tumor metastasis and recurrence, little is known about the underlying survival mechanisms in the blood circulation. The accumulating evidence has revealed that CTCs might survive in the peripheral blood by overcoming the mechanical damage due to shear stress, resistance to anoikis, evasion of immune destruction, and resistance to chemotherapy. The present review addresses the putative survival mechanisms underlying the formation and migration of CTCs according to their biological characteristics and blood microenvironment. In addition, the relationship between CTCs and microenvironment is illustrated, and the influencing factors related to the interactions of CTCs with various components in the peripheral blood are reviewed with respect to the platelets, immune cells, cytokines, and circulating tumor microemboli (CTM). Furthermore, the recent advances in the new treatment strategies targeting the survival mechanisms of CTCs are also discussed.

## 1. Introduction

**Figure 1:**
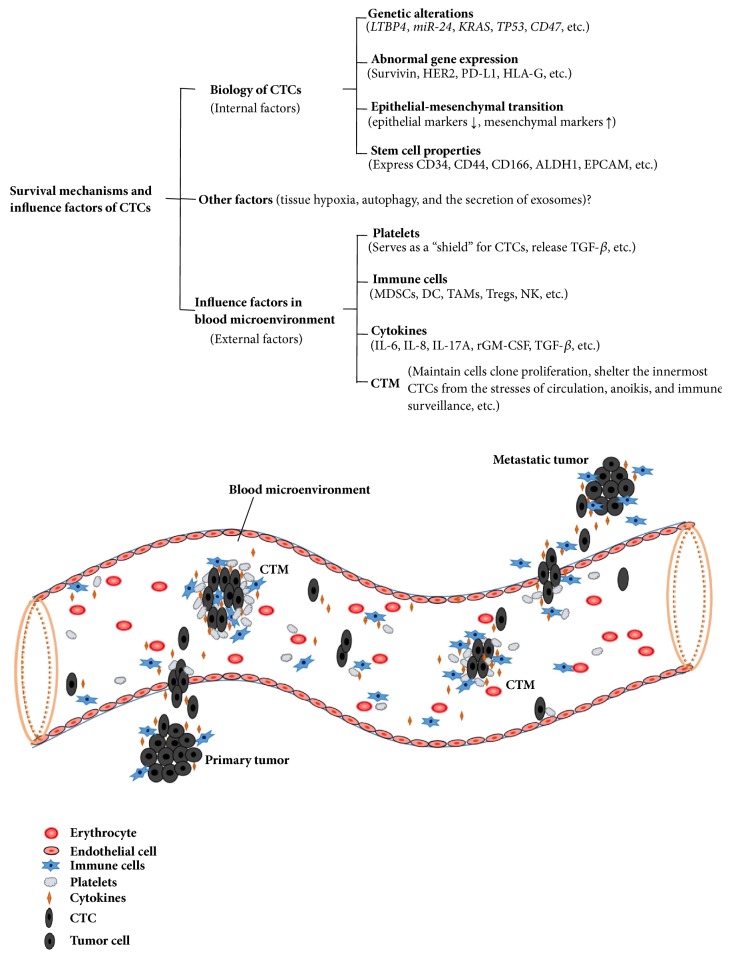
Survival mechanisms and influence factors of CTCs. This figure depicts the possible survival mechanisms underlying the formation and migration of CTCs in the blood microenvironment. The survival of CTCs is closely related to their internal biological characteristics, mainly including some genetic alterations, abnormal gene expression, epithelial-mesenchymal transition, and cancer stem cell properties. Moreover, various components in the peripheral blood, such as platelets, immune cells, cytokines, and CTMs, may interact with CTCs and promote their survival.

Recurrence or metastasis is the major cause of poor prognosis and mortality in patients with malignant tumor, >90% of cancer-associated deaths are caused by metastasis, and micrometastasis is the early event in the process of tumor metastasis [1, 2]. The presence of circulating tumor cells (CTCs) in the blood is closely related to tumor micrometastasis, which represents a poor prognosis in a variety of tumors. As a highly heterogeneous cell population, CTCs migrate away from a primary/metastasis tumor via the blood circulation to form secondary tumors in distant organs. They are considered as tumor micrometastasis biomarkers and real-time “liquid biopsy” sample, and the analysis of CTCs requires a blood sample that might provide an easy-to-repeat approach. A majority of CTCs exhibit anoikis or are subjected to the mechanical damage of shear stress in blood circulation. Only a small fraction of CTCs can survive, and <0.01% of these with high metastatic potential give rise to distant metastasis [2]. In recent years, accumulating studies focused on the survival mechanisms of the formation and migration process of CTCs based on their biological characteristics and blood microenvironment (see Figure 1); also, the effects on the mechanism of resistance to anoikis and evasion from immune destruction were emphasized. Herein, the potential survival mechanisms and related influencing factors of CTCs were reviewed, while the recent progress in the novel strategies for the treatment of tumor targeting the CTCs were also discussed.

## 2. Biology of CTCs

Currently, CTCs have been widely studied; however, the biology remains poorly understood. Furthermore, the release of CTCs from the primary/metastasis tumor, survival in blood circulation, circumventing apoptosis and host immune system (both innate and adaptive), and homing to distant organs is yet to be elucidated. Some studies showed genetic alterations or abnormal expression of some genes, and the physiological changes that affect the survival of CTCs in the peripheral blood that promote new distant metastases.

### 2.1. Genetic Alterations in CTCs

Genetic alteration is a major factor for tumorigenesis, and the presence of some survival-related gene changes in CTCs might be a crucial evolutionary mechanism that adapts to the external environment. The results of gene expression analysis of CTCs revealed a genetic variation as compared to the primary tumor cells, especially the apoptosis-related genes that are associated with the survival mechanism. Kanwar et al. performed high-resolution copy number profiling of CTCs from breast cancer to identify the changes occurring during the progression of the disease and found some genetic alterations in CTCs as compared to the primary tumor cells. These genes primarily involved the dormancy-related genes (*AKT2*,* PTEN*, and* CADM2*), invasion/metastasis-associated genes (*ANGPTL4*,* BSG*,* miR-373*, and* LTBP4*), and apoptosis-resistant genes (*miR-24*,* LTBP4*,* TFF3*,* NUMBL*, and* miR-181* family) [3]. Steinert et al. investigated a genomic analysis of single CTC from 31 individual patients with colorectal cancer, they exhibited some key genes of CTCs, such as* KRAS* and* TP53* that could not be detected in the corresponding tumor tissue; these genes are mainly involved in regulating cells proliferation/differentiation and apoptosis. Moreover, the pronounced upregulation of* CD47* gene that prevents the phagocytosis of macrophages and dendritic cells, suggesting a dormant state of viable CTCs, might be responsible for the survival of CTCs [4].

### 2.2. Abnormal Gene Expression of CTCs

In addition to genetic alterations, the differences in the molecular phenotype caused by the abnormal gene expression of CTCs might determine their biological effects. CTCs can express characteristic molecules, such as survivin, epidermal growth factor receptor, and immunosuppressive molecules, which may inhibit the apoptosis-related signaling pathways and promote the survival of CTCs.

#### 2.2.1. Survivin

Survivin is a major inhibitor of apoptosis protein that assists the escape of tumor cells from immune recognition by blocking the cytotoxicity of NK cells and inhibits the activation of apoptotic proteases, such as caspase-8 and caspase-6, thereby increasing the resistance of malignant cells to granzyme B and various chemotherapeutics [5]. Yie et al. found that the survivin mRNA was detected in the peripheral blood samples in 50.7% (34/67) breast cancer patients. During a follow-up period of 36 months, 81.8% (9/11) CTCs^survivin  (+)^ breast cancer patients suffered a relapse of the disease, whereas recurrence was only found in 33.3% (2/6) breast cancer patients with CTCs^survivin  (-)^ [6]. And more importantly, the similar results were verified in the further research of metastatic colorectal cancer (mCRC), esophageal squamous cell carcinoma and non-small-cell lung cancer, which revealed CTCs^survivin  (+)^ might be likely to survive during the migration of peripheral blood, and the detection of CTCs^survivin  (+)^ could provide a valuable prediction for recurrence and metastasis of malignancies [7–9].

#### 2.2.2. Epidermal Growth Factor Receptor

Human epidermal growth factor receptor 2 (HER2), also known as ErbB-2, is a member of the epidermal growth factor receptor family. It has tyrosine kinase activity and is closely related to the activation of multiple signal transduction pathways. Gene amplification/overexpression of HER2 is detected in several tumor cells, especially in CTCs, which play a crucial role in promoting tumor cell proliferation/growth, inhibiting apoptosis, and angiogenesis. The specific mechanisms underlying the CTCs expressing HER2 and promoting tumor cells survival in the blood circulation have not yet been fully elucidated. Supposedly, the expression of HER2 in CTCs could promote cell survival and proliferation through the PI3K/Akt and Ras/Raf/MAPK signaling pathway [10, 11]. Banys-Paluchowski et al. detected CTCs and serum HER2 (sHER2) levels using the CellSearch system and ELISA, respectively. They found that metastatic breast cancer (MBC) patients with ≥5 CTCs were more likely to present with elevated levels of sHER2 which were associated with worse survival [12]. Interestingly, Wallwiener et al. conducted a retrospective study and compared HER2 expression in primary tumors, metastatic tissue and CTCs from 107 MBC patients which revealed that primary and/or metastatic tumor tissue HER2-negative may CTCs HER2-positive, HER2 status can change during the course of breast cancer [13].

#### 2.2.3. Immunosuppressive Molecules

The upregulated secretion or expression of specific immunosuppressive molecules may be another survival mechanism of CTCs that escape from the surveillance of immune system and resist the cytotoxic effect of immune cells such as tumor antigen-specific T lymphocytes and NK cells. As a negative immune checkpoint regulator, the inhibitory costimulatory molecule of programmed death ligand 1 (PD-L1) plays a critical role in adaptive cellular immunity. The upregulated expression of PD-L1 on the surface of various types of cancer cells regulated the T-cell activation and differentiation and inhibition of the antitumor immune activity of T lymphocytes via specific binding with the receptor molecule of PD-1 on T-cells [14]. In order to identify the PD-L1 phenotype of CTCs, Mazel selected 16 individual HR^+^HER-2^−^ breast cancer patients with CTCs using the CellSearch system and for the first time found CTC^PD-L1(+)^ in 68.8% (11/16) patients. The detection rate of CTCs^PD-L1(+)^ varied from 0.2–100% and the PD-L1 expression of CTCs (based on the three rank immunoscoring method, 1–3) was divided into 1, 2, and 3 grades, which indicated a significant difference in the expression of PD-L1 on individual CTCs [15]. PD-L1 can mediate the regulatory T-cells (Tregs) to play a role in immunosuppression, and the interaction of PD-L1 with its receptor induces the apoptosis of activated T-cells [16]. In addition, CTCs^PD-L1(+)^-triggered PD-1 on T-cells may also increase the resistance of tumor cells to immune-induced death, suggesting that the CTCs might utilize the receptors on immune cells to induce resistance [17].

Similar to PD-L1, the expression of the human leukocyte antigen-G (HLA-G) has been observed in various malignant cancers. It is strongly associated with tumor immune escape, invasion/metastasis, and disease progression by inhibiting the immune cell cytolysis, proliferation/differentiation, and suppressing the cytokine production or inducing the apoptosis of immune cells, stimulating the generation of Tregs, and expanding the myeloid-derived suppressive cells (MDSCs) [18]. König et al. obtained the plasma samples from breast cancer patients before and after neoadjuvant chemotherapy; the samples were quantified for total soluble HLA-G (sHLA-G) and HLA-G levels by ELISA. The study demonstrated that high sHLA-G levels before neoadjuvant chemotherapy are closely correlated with disease progression, and the detection of stem cell-like CTCs (SL-CTCs) rendered them as major contributors to tumor immune escape and survival in the peripheral blood by expressing HLA-G or secreting sHLA-G [19].

### 2.3. Epithelial-Mesenchymal Transition (EMT) in CTCs

EMT in tumor cells is a highly complex dedifferentiation process, speculated to participate in the metastatic cascade, during which the epithelial markers such as cytokeratins, E-cadherin, and epithelial cell adhesion molecule (EpCAM) are downregulated, while the mesenchymal markers such as fibronectin, vimentin, and N-cadherin are upregulated [20]. The acquisition of EMT phenotype by tumor cells not only increases their invasive/migratory properties and decreases the adhesion characteristics, which enables them to migrate into the circulation and generate CTCs by traversing the basement membrane, interstitial spaces, and blood vessels but also promotes their survival in the blood microenvironment [21]. Compared to the primary tumor cells, CTCs underwent EMT that were more resistant against immune effector cells, anoikis, chemotherapy, and the mechanical shearing forces caused by blood flow. The phenotypic characterization of CTCs demonstrated the upregulation of EMT markers and their expression in specific tumor types [22]. Hanet al. investigated the correlation between the status of EMT of CTCs and esophageal squamous cell carcinoma, and found that 91.5% of them were epithelial-mesenchymal-mixed/mesenchymal CTCs, which were correlated with the number of total CTCs [23]. Ning et al. examined the prognostic significance of CTC EMT and stem cell gene expression by detecting the mRNA expression of EMT (PI3K*α*, Akt-2, Twist1) and stem cell (ALDH1) markers, and found that mCRC patients with CTCs expressing ALDH1, PI3K*α* and/or Akt-2 had a significantly inferior progression-free survival (PFS) and overall survival (OS) [24]. In addition to the formation of CTCs, EMT might also confer the antiapoptotic potential on CTCs. However, the specific molecular mechanisms are only slightly understood, leading to the speculation that the antiapoptotic potential may be related to the secretion of apoptosis inhibitors or activation of antiapoptotic signaling pathways in cancer cells that underwent EMT. Castillo et al. found that the emerging EMT (+) hepatocyte subpopulation secreted high levels of mitogenic and survival factors such as TGF-*ɑ* and heparin-binding EGF-like growth factor (HB-EGF), which activate the EGFR signaling pathway and TGF-*β*-induced resistance to the apoptosis signal [25]. Moreover, major EMT-related transcription factors such as Snail and Twist could also resist apoptosis through a variety of mechanisms. Some researches proved the Snail-mediated EMT which can inhibit the apoptosis of MDCK cells by downregulating the expression of caspases and Spermatogenic Zip 1 (SPZ1)-Twist complex could act as a protooncogene, and improve VEGF expression via the recruitment of BRD4, thus promoting RNA-Pol II-dependent transcription and inducing tumor metastasis [26, 27].

### 2.4. Stem Cell Properties of CTCs

Cancer stem cells (CSCs) are special types of cell populations in tumors with stem cell properties such as self-renewal, unlimited proliferation, and indirect differentiation potential [28]. Few CTCs that possess stem cell properties in the peripheral blood are termed as circulating tumor stem cells (CTSCs). Such cells represent the critical population that predicts tumor progression and monitors the curative effect. An increasing number of studies confirmed that the formation of CTSCs is closely related to the occurrence of EMT; CTCs can undergo EMT and assume a stem cell-like phenotype. The change in some genotypes (*NANOG*,* OCT3/4*,* SOX2*) and molecular phenotypes (ICAM-1 and CD133) of CTCs confer the stem cell features, which promote the survival of CTCs [29, 30]. Specific molecules including CD34, CD44, CD166, ALDH1, and EpCAM that are expressed in a variety of tumor cells are considered as the primary biomarkers for the identification of CSCs. Li et al. investigated the prognostic significance of stem cell-like CTCs in gastric cancer patients, and found that detected 70.4% (19/27) with CD44 (+) CTCs. The results indicated the proneness of these patients to develop metastasis/recurrence than those with CD44 (-) CTCs, thereby indicating that the CTSCs might provide a clinically valuable prognosis than only CTCs [31]. Moreover, Theodoropoulos et al. reported the occurrence of a subpopulation of CTCs with stem cell-like phenotypes in patients with metastatic breast cancer. Among a total of 1439, CTCs were detected in 66.7% (20/30) patients and 35.2% exhibited the stem-like phenotype CD44^+^/CD24^-/low^, whereas 17.7% of the CTCs were ALDH1^high^/CD24^-/low^ [32]. Since CTCs expressed a variety of CSCs markers involved in a decreased sensitivity to chemotherapeutics and cytotoxic immune effector cells, the CTSCs are speculated to exert resistant to the immune system and survive in the peripheral blood as compared to CSCs markers-negative CTCs [30].

Metastasis starts with CTCs release from the primary tumor through the surrounding tissue into the bloodstream. CTCs migration (mobility and motility) may be acquired by different processes of which EMT and their internal biological characteristic mentioned above are currently believed to be the main ones. The alteration of some genes and the abnormal expression of some molecules in CTCs endow them with the potential of abnormal proliferation and resistance to apoptosis. EMT increases cell plasticity and provides a prerequisite for metastasis of primary tumor in the process of CTCs migration. Actually, CTCs survival is a complex process involving multiple factors. It is not only associated with their specific characteristics but also closely related to the hostile microenvironment in the blood. In addition to the visible components such as platelets, various immune cells and the intangible components including cytokines also exist in the bloodstream, and the change in these two components and their interaction with CTCs play a critical role in the anoikis resistance and immune escape of CTCs. Next, we elaborated several factors in blood microenvironment such as platelets, immune cells, cytokines, and circulating tumor microemboli (CTM) and discussed their effects on the survival mechanisms of CTCs.

## 3. Survival Mechanisms of CTCs in Blood Microenvironment

### 3.1. Platelets

The metastasis and prognosis of tumor are highly influenced by the number and activation of platelets, which promote the survival of CTCs during metastasis by conferring resistance to the shear stress and attack from NK cells [33, 34]. Interestingly, some evidence suggested that the use of antiplatelet agents and anticoagulant therapy promotes CTCs survival [35, 36]. To investigate the mechanisms of platelet activation in tumor growth and dissemination, Palumbo et al. studied metastasis in mice lacking Galphaqas compared to the formation of lung metastatic node in control group mice and Galphaq-deficient mice following the intravenous injection of either lung carcinoma cells or melanoma cells. The results indicated that Galphaq deficiency significantly reduced the metastatic potential of CTCs [37]. Specifically, the obvious reduction in the survival of CTCs observed in fibrinogen/Galphaq-deficient mice as compared to the control group was eliminated by NK cells. Furthermore, the expression of some molecules on the surface of platelets and the release of cytokines might also contribute to the survival of CTCs in the bloodstream. Supposedly, the platelet-derived TGF-*β* might inhibit the protective host immunity and promote the escape of CTCs from immune clearance via multiple mechanisms: (1) TGF-*β* exerts diverse effects on a variety of cellular processes including cell proliferation, differentiation, and activation, as well as the apoptosis of immune cells induced by downregulating the expression of antiapoptotic Bcl-2 proteins [38]; (2) TGF-*β* mediates the downregulation of NKG2D expression and might be responsible for impaired cytotoxicity of the NK cells [39]; (3) TGF-*β* released from the activated platelets directly counteracts NK cells and CTLs granzyme mobilization, cytotoxicity, and perforin/interferon-*γ* secretion [34, 40]. Notably, the expression of fibrinogen receptor GPIIb-IIIa (*α*IIb*β*3 integrin) and P-selectin on the platelets mediate the attachment of platelets and CTCs that express the membrane surface molecules such as *α*v*β*3 integrin and the cell adhesion molecule CD44, indicating a robust correlation with tumor dissemination [34, 36, 37]. Furthermore, the platelet-CTC interaction can lead to the transfer of platelet MHC-I to tumor cells, thereby preventing the identification of NK cells and aiding the CTCs to escape from the cytolytic activity mediated by NK cells [41]. On the other hand, the aggregation of platelets serves as a “shield” for tumor cells in the circulation, enabling the formation of CTC-platelet complexes and evading immune clearance [37].

### 3.2. Immune Cells

A principal reason for the nonsurvival of a majority of CTCs is their potential to be eliminated by the immune cells. However, few CTCs can escape immune surveillance with the help of other types of immune cells in the circulating blood, effectuated by a complicated mechanism. In addition to promoting the formation of the local immunosuppressive microenvironment in tumor tissue, the circulating immune cells, especially the MDSCs equipped with immunosuppressive effects might protect the CTCs from antitumor immune attack by coating them forming cell clusters. Although the hypothesis that immune cells could aid the CTCs escape from host immunity has not yet been directly validated, the existing evidence that immune cells adhere to some cancer cells and form tumor cells-lymphocyte chimeras in various malignant tumors indirectly substantiated the theory [42]. Moreover, immune cells in the peripheral blood may be involved in the immune escape of CTCs by altered molecular phenotype. Gruber et al. found that the CD95L/FasL (ligand molecule) expressed on CTCs in patients with breast cancer interacted with the upregulated transmembrane receptor CD95 (APO-1/FAS) on the surface of peripheral T-helper (Th) cells, which might mediate the transmission of apoptosis signal, contribute to systemic immunosuppression, and lead to the dormancy of CTCs [43]. In a recent study, Green et al. detected the expression of Toll-like receptors (TLRs) on the surface of dendritic cells in peripheral blood, which are thevital members of the innate immune system with a critical role in antitumor immunity [44]. Flow cytometry showed a markedly increased expression of TLRs in CTCs (+) patients as compared to CTCs (-) patients, and the number was correlated to the expression. The sustained activation of TLR signaling pathway might promote tumor cells proliferation and immune evasion through the release of inflammatory cytokines, chemokines, and growth factors, thereby creating a suppressive microenvironment for the survival of CTCs. Additional studies are essential to further investigate the other immune cells with an inhibitory function such as tumor-associated macrophages (TAMs) and Tregs and their role in promoting the immune escape of CTCs.

### 3.3. Cytokines

Previous studies have demonstrated that the prognosis of CTCs is closely related to various types of cytokines. A study by Kim et al. in breast carcinoma and melanoma models showed that cytokines such as interleukin-6 (IL-6) and interleukin-8 (IL-8) might be tumor-derived attractants of CTCs, which could induce the clonal expansion of CTCs in distant organs as well as attract the CTCs back to a primary tumor; this phenomenon is termed as “tumor self-seeding by CTCs” [45, 46]. As a multifunctional proinflammatory cytokine, IL-6 is expressed in pathological samples obtained from patients with malignant tumor and in multiple cell lines. It can suppress the tumor cell apoptosis through the PI3K/Akt signaling pathway and regulate the downstream signaling molecular cyclin A1, which is an essential factor in the prosurvival network [46, 47]. IL-8 is another vital molecule involved in tumor cell apoptosis. Xiao et al. confirmed that IL-8 expression was negatively correlated with anoikis in colorectal cancer (CRC) cells. IL-8 treatment improved the resistance of tumor cells to apoptosis with the increased level of T-LAK cell-originated protein kinase (TOPK) by activating the PI3K-Akt and Ras-Raf-Mek-Erk signaling pathways [48]. Another study conducted by Tseng et al. demonstrated a positive/negative correlation between IL-17A/granulocyte-macrophage colony-stimulating factor (GM-CSF) and the frequency of CTCs by continuously monitoring the dynamic changes in cytokines levels and CTCs in serum. In addition, the ablation of IL-17A and rGM-CSF treatment caused a decline in the number of CTCs and decreased metastasis in mice. Furthermore, the specific mechanism affecting the survival of CTCs was also confirmed. The presence of IL-17A promoted the formation of CTCs and motility by inducing the expression of matrix metallopeptidase 9 (MMP-9) in tumor cells, matrix degradation, and angiogenesis, whereas GM-CSF administration polarized the TAMs toward the M1 phenotype (an antitumor type), increased the M1/M2 (a protumor type) ratio, and thus, stimulated the elimination of CTCs by elevated number of CD4^+^ and CD8^+^ T lymphocytes and NK cells [49]. In addition to the above exemplary proinflammatory cytokines, some immunosuppressive cytokines such as IL-10, IL-35 and TGF-*β* might also inhibit the immune cells function and prevent the elimination of CTCs.

### 3.4. CTM

In the past decade, increasing number of studies provided evidence that CTCs can be present in the bloodstream in multiple forms such as solitary cells and cell clusters; thus, CTM may be composed of at least 2 CTCs with large microemboli containing >50 CTCs [50, 51]. The formation of CTM might result either from the intravasation of cancer cell clumps via vessels and lymphatic ducts in the primary tumor or from their aggregation in the blood circulation due to the collective migration and adhesion [52]. In contrast to simple CTC, CTM shows a high potential of metastasis by maintaining cell clonal proliferation, protecting the innermost cells from the stresses of circulation, anoikis, and immune surveillance [50]. These characteristics indicate that the analysis of these CTCs provide a comprehensive and accurate information for the prognosis and treatment of patients with advanced cancer. CTM are composed of CTCs and other components such as leukocytes, cancer-associated fibroblasts, endothelial cells, and platelets that provide a favorable microenvironment for crosstalk and survival [51, 53]. Intriguingly, CTM can shield the CTCs from host immune attack by forming an effective physical barrier on the surface of tumor cells in circulation. On the other hand, a variety of nontumor components that constitute CTM may interact with each other and promote the proliferation of cancer cells by inhibition of apoptosis while releasing several kinds of cytokines, including platelet P-selectin, von Willebrand factor (vWF), and the tissue factor (TF). Although the role of CTM in tumor metastasis has been emphasized for a prolonged period, the differences between CTM and CTCs were studied recently. Hou et al. demonstrated significant differences in the biological behavior of CTCs and CTM, including that of the apoptosis and proliferation status. The study analyzed blood samples from 97 patients with small-cell lung cancer receiving chemotherapy. None of the cells comprised of CTM exhibited apoptotic nuclear morphology, even in patients with apoptotic solitary CTCs, and overexpression of the antiapoptotic protein Bcl-2 in CTM indicated their survival in the circulating blood. Moreover, the proliferation status of simple CTCs and CTM was also assessed by detecting the expression of Ki67 proliferation nuclear antigen using the isolation by size of epithelial tumor cells (ISET) filter technique. The results showed Ki67 expression was detected in individual CTCs from blood specimens (n=20) in variable proportions of cells, which present a sharp contrast with all CTMs (n=34) were negative expression for Ki67, even in patients with Ki67 (+) CTCs [35]. Thus, in comparison with CTCs, CTM might remain inactive (dormant) in an optimal environment by inhibiting the proliferation and apoptosis.

The dissemination of CTCs through the blood circulation is an important intermediate step that also exemplifies the switch from localized to systemic disease. Therefore, investigating how to prevent CTCs from metastatic spread is of vital interest to tumor biology and a deeper understanding of what factors allow CTCs to survive in the blood microenvironment could be used to find out new therapeutic targets.

## 4. New Treatment Strategies Targeting the Survival Mechanisms of CTCs

The further study about the physiological characteristics of CTCs and the mechanism of varieties of factors in promoting the survival of CTCs in peripheral blood can aid in selecting an appropriate clinical treatment for different cancer patients. The anticoagulant therapy used in the cancer patients and animal models can effectively reduce the risk of tumor metastasis. Since the platelets can promote the survival and metastasis of CTCs in blood, and the anticoagulant therapy may cause bleeding and other adverse reactions. Li et al. explored the genetically modified platelets that express tumor necrosis factor-related apoptosis-inducing ligand (TRAIL) and found that the blockade of the platelets-CTCs interactions could serve as an effective antimetastatic strategy in a model of prostate cancer metastasis. Moreover, the TRAIL-expressing platelets could significantly eradicate the tumor cells in vitro [35]. The recent studies found that the newly emerging nanotechnology has broad prospects in CTCs' research and could be a promising approach to kill CTCs with a high efficiency. The nanoparticles combined with apoptosis-related molecules such as TRAIL, TNF, FasL, and liposomes with encapsulated siRNA will prolong the overall survival of the patients with malignancy [35, 54]. In addition, some mutated or abnormally expressed genes associated with CTCs survival may be new targets for cancer therapy. Thus, the individualized clinical treatment of anti-EGFR/KRAS in patients with colorectal cancer is based on the analysis of* KRAS* gene mutation in CTCs [4, 55]. Furthermore, the detection of changes in the expression of* HER2* gene in CTCs might also contribute to the stratification of breast cancer patients and develop effective individual therapeutic regimen targeting the* HER2 *gene. This suggested that the gastric cancer and MBC patients with HER2-negative in primary and (or) metastatic tumors, but HER2-positive in CTCs might benefit from Trastuzumab, a promising HER2-targeted drug that inhibits the growth and proliferation of malignant tumor cells [13, 56]. Notably, the study by Dondossola et al. found that CTCs with “tumor self-seeding” property could promote the development of novel targeted cancer therapy. The study genetically engineered a variety of tumor cells such as mouse mammary adenocarcinoma (TSA), Lewis lung cancer cells (LLC), and melanoma cells (B16-F10) to produce and release the potent antitumor cytokine TNF. Subsequently, these cells were intravenously injected into mice as CTCs, which demonstrated that the genetically modified CTCs could home to the primary tumors, locally release TNF, damage the tumor angiogenesis, and induce tumor cell apoptosis, thereby inhibiting tumor growth. Thus, CTCs could serve as potential carriers for antitumor agents [57].

## 5. Conclusions

The survival of CTCs in the blood circulation is a complex process, involving various factors and several mechanisms. In addition to the influencing factors described above, tissue hypoxia, autophagy, and the secretion of exosomes might also affect the outcome of CTCs. However, the current understanding about the survival mechanisms underlying CTCs is limited and is based on experimental and theoretical extrapolations. The clinical classification according to the molecular phenotype/karyotype of CTCs and individualized treatment options according to the antiapoptosis mechanisms of CTCs are yet to be elucidated. Therefore, further studies investigating the physiological characteristics of CTCs and their survival mechanisms in the blood microenvironment would be remarkable in seeking for new therapeutic targets and optimizing the clinical treatment strategies, thereby contributing to design novel therapeutic treatments.
